# The chosen few—variations in common and rare soil bacteria across biomes

**DOI:** 10.1038/s41396-021-00981-3

**Published:** 2021-05-25

**Authors:** Samuel Bickel, Dani Or

**Affiliations:** 1 Institute of Biogeochemistry and Pollutant Dynamics, ETH Zurich, Zurich, 8092 Switzerland; 2 Institute of Terrestrial Ecosystems, ETH Zurich, Zurich, 8092 Switzerland; 3 Division of Hydrologic Sciences, Desert Research Institute, Reno, NV USA

**Keywords:** Biodiversity, Microbial ecology, Microbial ecology, Soil microbiology

## Abstract

Soil bacterial communities are dominated by a few abundant species, while their richness is associated with rare species with largely unknown ecological roles and biogeography. Analyses of previously published soil bacterial community data using a novel classification of common and rare bacteria indicate that only 0.4% of bacterial species can be considered common and are prevalent across biomes. The remaining bacterial species designated as rare are endemic with low relative abundances. Observations coupled with mechanistic models highlight the central role of soil wetness in shaping bacterial rarity. An individual-based model reveals systematic shifts in community composition induced by low carbon inputs in drier soils that deprive common species of exhibiting physiological advantages relative to other species. We find that only a “chosen few” common species shape bacterial communities across biomes; however, their contributions are curtailed in resource-limited environments where a larger number of rare species constitutes the soil microbiome.

## Introduction

Bacterial communities are characterized by strongly skewed relative abundance distributions (RADs) with most phylotypes (or “species” for simplicity) present at low relative abundances [1]. These rare bacterial species are considered ecologically important [2]. They contribute to the vast richness of prokaryotic taxa that supports functional diversity [3–5] and specific ecosystem functions [6–10]. Despite the high functional potential of the soil microbiome and its diverse genetic composition [5, 11], only a few bacterial species appear to be prevalent across soils from different environments [12, 13]. This raises questions regarding the role of the vast majority of soil bacterial species and their contribution to ecosystem functioning and resilience [4, 6–9, 14]. Evidence suggests that rare bacterial species contribute to specific functional traits [3, 5, 15] and exhibit greater sensitivity to environmental factors than common species [16–18]. However, the processes that affect rare bacterial species remain largely unknown [16, 19, 20] or are overlooked [21].

Despite a growing interest in the “rare biosphere” and empirical patterns of microbial rarity, methods for distinguishing globally common and rare soil bacteria are lacking [12, 15]. In many studies, rare bacterial species are ignored [2], particularly in global studies that focus on the relative abundance patterns of common species [13]. Operationally, the classification of common and rare bacterial species is based on prevalence or relative abundance. Prevalence measures the probability of detecting a species across samples, while relative abundance measures the probability of encountering a species within a soil sample (~1 cm^3^). Both aspects are important for assessing how likely it is to find a bacterial species (or group of species) in soils and for linking species prevalence and abundance with ecosystem functioning [12, 22, 23].

The conditions under which rare species contribute substantially to ecosystem services remain understudied across the entire tree of life [24]. In contrast, the broad fitness of common bacterial species [16, 23] promotes their success across a wide range of environmental conditions [18]. Consequently, common bacterial species are often poor indicators for changes in environmental conditions and community composition [21, 25]. Furthermore, we expect species proportions to be biased by the physiological state of bacteria [21] because a large proportion of cells in soil could be dormant or inactive [10]. To better assess compositional changes in the soil microbiome and properly attribute the contributions of rare species to ecological functioning, we need a universal classification of common and rare bacteria.

In this study, we seek to understand what determines the proportions of common and rare soil bacterial species across biomes by (i) developing a universal metric for global classification of rare and common soil bacteria based on relative abundance and prevalence, (ii) identifying patterns of richness and abundance with environmental conditions, and (iii) employing a mechanistic model to quantify how climatic factors shape the proportions of rare soil bacteria.

**Fig. 1:**
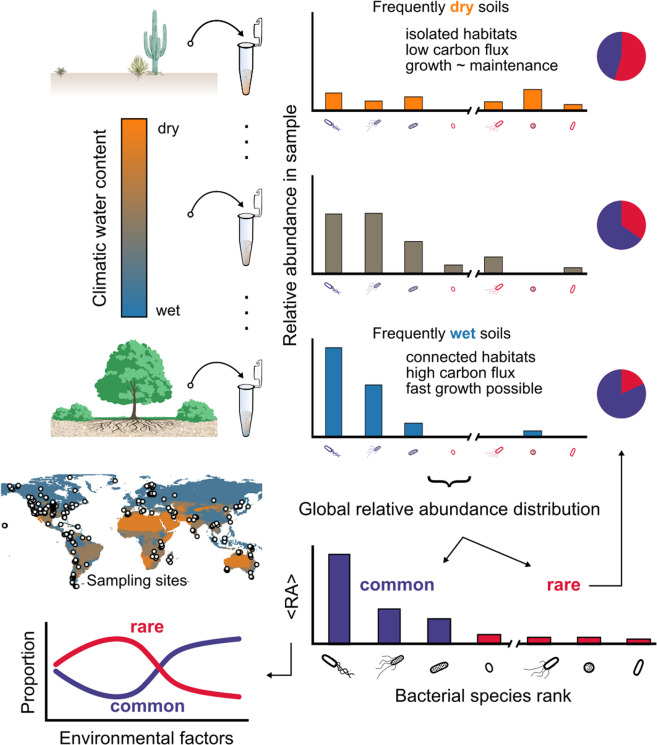
Tracking globally common and rare soil bacteria across environmental conditions. We perform analysis of a global pool of bacterial relative abundance distributions (RADs) from soil samples across biomes with a wide range of climatic water contents. Bacterial species relative abundance (RA) from individual samples is used to obtain the global, average RAD, and species ranking. Each species is classified as common or rare based on the global RAD. The proportions of common and rare bacteria are tracked across environmental factors. A particular focus lies on shifts in proportions with a climatic water content that is a proxy for a soil’s aqueous-phase connectedness. Dry soils are expected to host communities with a higher evenness that include many rare species by physically limiting growth to isolated aqueous habitats with low carbon fluxes. In frequently wet soils, a “chosen few” common species are expected to grow rapidly and dominate the soil bacterial community.

The study is motivated by statistical models that demonstrate increasing explanatory power of environmental variables for bacterial diversity when additional weight is given to locally low abundant species [17]. These species are expected to be sensitive to environmental factors with unique biogeographic patterns that might reflect distinct ecological strategies (e.g., *r* and *K*) [11]. We perform a global analysis of previously collected [17] and sequenced soil samples [26–28] and classify soil bacterial species as common or rare based on the pooled RAD from all sampled soils across the globe. The obtained proportions of rare and common bacterial species in individual soil samples are investigated with regard to key environmental variables (Fig. 1): mainly the climatic water content [29] (CWC), the net primary productivity (NPP), and mean annual temperature (MAT). Evidence suggests that CWC plays a crucial ecological role in promoting soil bacterial community diversity by the intrinsic fragmentation (or connectedness) of microscale aqueous habitats [17, 29]. High values of CWC (wetter soils) also support higher vegetation density and increase carbon inputs that enhance soil-carrying capacity and total bacterial biomass [29–31]. At the microscale, these resource fluxes are spatially distributed and shape bacterial life on soil grain surfaces (~1 mm^2^). Here, we employed a spatially explicit individual-based model (SIM) of soil microbial communities to understand the mechanisms that generate patterns of commonness and rarity under conditions that are characteristic of terrestrial biomes. We hypothesize that drier soils with highly fragmented aqueous habitats and restricted diffusion of carbon suppress the activity of fast-growing common bacteria and lead to communities with more even RADs [32].

## Materials and methods

### Soil bacterial community data

A global dataset on soil bacterial community composition across biomes was used to delineate patterns of common and rare soil bacteria. Only samples originating from natural soil environments were considered in the previously published dataset[17] []. The georeferenced samples (*n* = 844) were aggregated to 318 sites at a spatial resolution of 0.1° for evaluating the effects of environmental drivers on bacterial rarity. Most major biomes were represented by *n* = 21–46 samples, except tropical grasslands, tropical forests, and temperate forests that were overrepresented with *n* = 113, 272, and 260 samples, respectively.

The detailed methodology used to combine the raw (16S rRNA V4) sequence data of soil samples from three studies [26–28] was previously described [17]. Briefly, the sequences were dereplicated and denoised after trimming to 90-bp length. Singletons were removed before denoising for each sample, resulting in a total of 256 620 unique amplicon sequence variants (ASV) of which 71% were observed less than ten times across samples [17]. ASVs were assigned taxonomy using a multinomial Naive Bayes classifier trained on Greengenes 13_8, 99% OTUs (515F–806R region). Sequences that could not be classified confidently (<70%) as bacteria at the Kingdom level or sequences classified as archaea were discarded. In addition, global singletons (observed only once across samples) were removed.

The resulting table of ASV abundance (referred to as “species” abundance) was then rarefied to a total count of *N* = 7 544 per sample. In this study, the independent rarefication was averaged for 15 realizations. This was sufficient to achieve a robust classification of common and rare bacteria using the methods outlined below. The prevalence of each species was estimated as the number of nonzero rarefied counts $c$ divided by the number of samples $n$. For every sample $k$, the relative abundance (proportion) $p$ was obtained by dividing counts of species $i$ by the total counts after rarefication as $p_{i,k} = \frac{{c_{i,k}}}{N}$. Subsequently, we obtained the global relative abundance $g$ for each species by averaging across samples according to $g_i = \frac{1}{n}\mathop {\sum}\nolimits_{k = 1}^n {p_{i,k}}$. We thus distinguished the local (e.g., sample) RAD from the global RAD that was subsequently used for the classification of common and rare soil bacteria.

### Classification of common and rare bacteria

An algorithm for automatic threshold selection based on minimizing cross-entropy [33] was used to designate common and rare bacteria using only the global RAD. The algorithm was originally developed for image segmentation and was previously implemented (function “threshold_li” in scikit-image 0.14.0 [34]). This approach makes no a priori assumption on the underlying distribution of values and provides an unbiased estimate of the binary classification [33]. Cross-entropy has been used before to estimate rare event probabilities [35] and more recently to classify sequences for taxonomic assignment to address issues of overclassification in the presence of novel groups [36]. Here we used the obtained threshold value *t* to distinguish common and rare species based on each species global relative abundance $g_i$. The species with $S_{\textrm{{r}}}=\{ i|g_i \le t\}$ were considered “rare” and species with $S_{\textrm{{c}}}=\{ i|g_i \,> \, t\}$ were defined as “common”. The relative abundance of rare (${\mathrm{RA}}_{\mathrm{r}}$) and common (${\mathrm{RA}}_{\mathrm{c}}$) species in a single sample was thus given by ${\mathrm{RA}}_{{\mathrm{r}},k} = \mathop {\sum}\nolimits_{i \in S_r} {p_{i,k}}$ and ${\mathrm{RA}}_{{\mathrm{c}},k} = \mathop {\sum}\nolimits_{i \in S_c} {p_{i,k}}$ for proportions of rare and common species, respectively. The general classification introduced here was applied to a global soil community DNA dataset [17] and to a previously published soil community RNA time series [37].

### Climatic data of sampling locations

Environmental variables for each topsoil (≤10 cm) sample were added at their highest native resolution based on latitude and longitude using nearest-neighbor interpolation as previously reported [17]. NPP (MODIS [38], averaged for 2000–2015) and MAT (WorldClim [39]) were used to estimate maximal cell density (potential-carrying capacity) as previously described [29] by dividing the soil carbon input flux by a temperature-dependent [40] maintenance rate that is specific to biomass carbon (≈10^−4^ gC gC^−1^ h^−1^). For soil wetness, we used CWC as a proxy for climatic soil hydration conditions and soil aqueous-phase connectivity [17, 29]. Values were based on global gridded precipitation time series (MSWEP [41], daily for 1979–2016 at 0.1° spatial resolution) that yielded the average number of consecutive dry days $\tau$ used for the calculation of CWC [17]. Rainfall frequency was obtained by taking the inverse ($\tau ^{ - 1}$). Due to their central role, estimates of CWC were also compared to mean soil moisture obtained from climate model reanalysis (ECMWF ERA5-Land, 0–7 cm, monthly for 1981–2019 at 0.1° spatial resolution, 10.24381/cds.68d2bb30). Both climatic soil moisture estimates were in good agreement (*n* = 318, slope = 0.996, intercept = 0.066, and *R*^2^ = 0.54), despite their methodological differences and other uncertainties associated with remote sensing data in general. We thus did not expect considerable bias in patterns of bacterial rarity by the choice of climatic soil moisture estimates.

### Spatially explicit individual-based model (SIM)

An individual-based model was used to simulate the growth of diverse bacterial species on soil surfaces [29, 42, 43]. Briefly, the model considers a heterogeneous surface in the pore space of a defined soil volume (specified by 1-mm^2^ area and 11-µm thickness of a soil slab). We simulated continuous growth and movement of individual cells on the two-dimensional domain with shared resources. Three diffusible carbon sources were modeled that could be consumed at rates bounded by cellular capacity [44]. Different bacterial species were represented by unique combinations of kinetic parameters reflecting their competitive ability under locally variable carbon source concentrations. For simplicity, all the kinetic parameters were assigned the same temperature dependency [40]. All simulations were initialized with the same total number of species assuming no dispersal limitation at initial conditions. From each of the modeled 3 360 species, a single cell was initially placed at a random location. The simulation time was eight days with a time step of one minute and with nutrients replenished, on average, every 4 h. This enabled a maximum cell density of around 10^17^ cells per m^3^ of soil and the simulated time was sufficiently long for the communities to stabilize. The prescribed generation times under ideal conditions ranged from 0.6 h to 288 days. The time between nutrient pulses corresponded to a potential maximum of 2.4 generations. At the end of the simulations, cells of each species were counted to obtain the RAD. Predictions of the SIM were evaluated for a range of soil moisture conditions that shape the diffusion of nutrients and the mobility of cells on hydrated soil surfaces [42, 43]. All interactions among cells emerged from their relative spatial positioning within the nutrient field and their species-specific carbon utilization patterns. For details of the implementation and a summary of trait parameters see Supplementary Methods and Table S1, respectively.

## Results

### Relative abundance and prevalence of common and rare soil bacteria

**Fig. 2:**
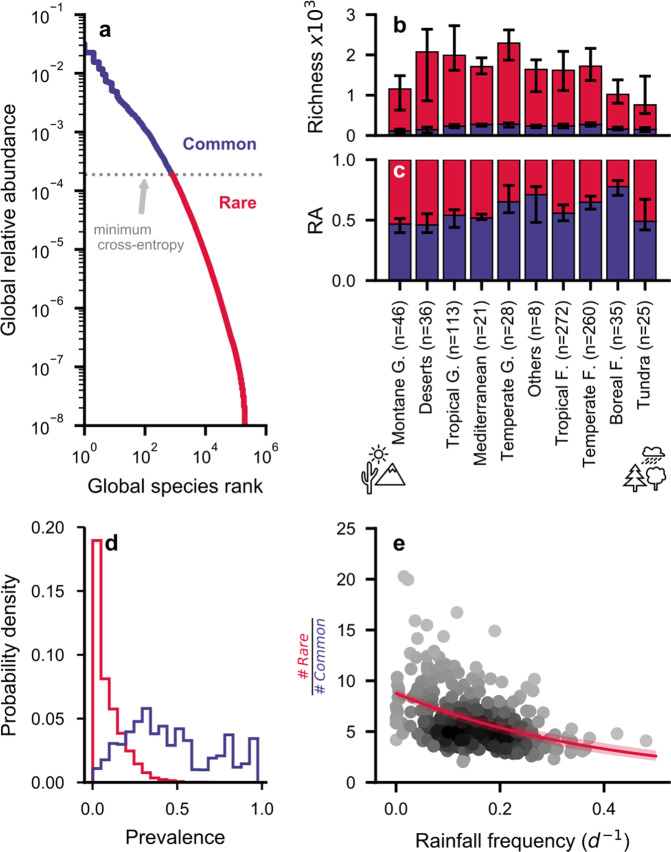
Observed proportion and richness of common and rare bacteria across biomes. **a** Global relative abundance distribution (RAD) of bacterial species (*n* = 844). The dashed line indicates the threshold based on minimum cross-entropy [33] that distinguishes common and rare species using only the global RAD (shown in blue and red, respectively). **b**, **c** Bacterial richness and relative abundance (RA) for common and rare bacteria vary across biomes. Stacked bars indicate the median ± IQR. The number of samples for each biome is reported (G = grassland, F = forest). **b** Richness of rare bacteria varies more strongly among biomes compared to common bacteria, while, **c** their RA seldom exceeds the RA of common species. **d** Prevalence, the fraction of samples in which a species occurs, is related to the global RAD. **e** The ratio of rare-to-common species declines with increasing rainfall frequency for different sampling sites (exponential *R*^2^ = 0.19; Pearson *r* = −0.41, *n* = 318).

We have used previously published [17] genomic data (16S rRNA gene sequences) from soil samples [26–28] (*n* = 844) across major biomes to identify global patterns of common and rare bacterial “species” (90-bp rRNA ASV). The classification of common and rare species was achieved by using a global threshold of relative abundance based on minimizing cross-entropy [33], i.e., a threshold that minimizes the amount of information needed to reconstruct the RAD given the binary classification of common and rare species (Fig. 2a). The resulting threshold to delineate the relative abundance of common species was remarkably consistent (0.019 ± 0.002%, bootstrap mean ± SD; Table S2) and comparable to previous, empirical, or operationally defined thresholds based on relative abundance [12, 17, 20]. Most bacterial species were classified as rare (99.6%) and made up only 42% of the global relative abundance. The threshold selection resulted in average proportions of rare species that were robust even when using ¼ of all samples available (Table S2). With low sample numbers, fewer rare species were included and the selected thresholds increased slightly. This was not unexpected since species with lower abundance were better represented with more samples, resulting in overall lower thresholds for classification. To test for potential sampling bias due to the underrepresentation of biomes, we resampled RADs from each biome with replacement (*n* = 21, repeated 50 times) and obtained threshold values (0.024 ± 0.003%) comparable to those obtained from small datasets (Table S2). Thus, we did not detect considerable bias in patterns of rarity.

Soil bacterial community richness and the cumulative relative abundance of rare and common species varied among biomes indicating sensitivity to environmental conditions (Fig. 2b, c). Generally, common species with high relative abundance were more prevalent than rare species (Fig. 2d). The average prevalence (median ± IQR) for common species (0.3 ± 0.2) was 300 times larger than for rare species (0.001 ± 0.003). Besides, the ratio of rare species richness to common species richness decreased significantly with more frequent rainfall (exponential *R*^2^ = 0.19, Pearson *r* = −0.41, *n* = 318), indicating that community composition may vary with the climatic soil water content (Fig. 2e). We found that dry ecosystems hosted diverse and highly variable communities that were different from the globally expected RAD (Fig. S1). A large number of rare and endemic species were observed in dry soils, while few common species dominated wetter soils that were better represented by the average global community composition.

### The rarity of soil bacterial species is shaped by CWC

**Fig. 3:**
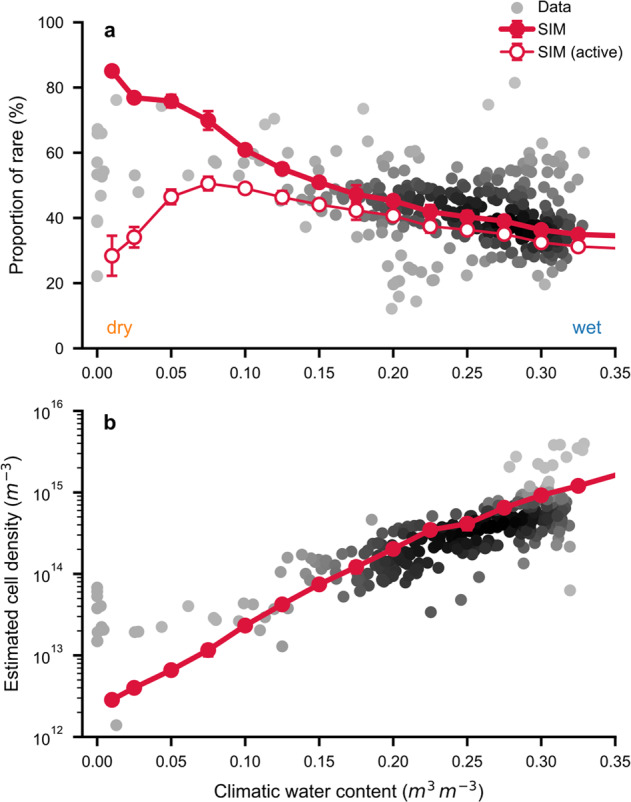
Decline in observed soil bacterial rarity is mechanistically linked with hydration conditions and carrying capacity. **a** The proportion of rare bacteria decreases with increasing climatic water contents (Spearman *ρ* = −0.36, *n* = 318; highest density of points shown in black). The decline in rare species proportion is predicted by a spatially explicit individual-based model (SIM, mean ± SD, *n* = 5) and compares favorably with empirical observations. Considering only active cells by removing cells that did not divide in the results of the SIM causes a drop in rare proportion under dry conditions (open symbols). **b** Estimated cell density (potential carrying capacity) increases exponentially with climatic water contents. Cell density is calculated based on mean annual temperature, carbon input flux (net primary productivity), and bacterial maintenance requirements using independent data [29, 30]. The prediction by the SIM makes no assumptions on the relation of cell density with soil hydration conditions.

Accompanying a climatic transition from wet to dry soils, we observed a gradual shift in RADs toward more even bacterial communities in climatically drier soils (Fig. S2). To understand the mechanisms and drivers for these changes in soil bacterial rarity, we used the mechanistic SIM that makes no assumptions regarding species composition or each species relation to soil moisture conditions. Model results show that only a few common species dominated bacterial communities under wet conditions (Fig. 3a). To test how the proportions of rare bacteria were affected by the presence of cells with reduced activity (dormant or at maintenance rate state), we removed cells that did not divide during the simulated timespan from the modeling results. The removal of these inactive cells resulted in a sharp decrease in the modeled proportion of rare species under very dry conditions (Fig. 3a).

Overall, bacterial cell density increased significantly under wet climatic conditions with enhanced carbon fluxes as seen in model simulations and empirical estimates of maximal cell density (Fig. 3b). This carrying capacity was estimated from carbon input by NPP and mean maintenance requirements of soil bacteria (adjusted for MAT) with no explicit dependency on CWC [29]. Considered independently, these two factors (NPP or MAT) did not exhibit clear tendencies for changes in proportions of common and rare bacterial species (Fig. S3). We examined the effects of temperature using the SIM with temperature-dependent bacterial growth [40, 45]. Biome-specific CWC and MAT were used as boundary conditions for comparison with data to highlight the predominant influence of soil moisture on soil-carrying capacity and the proportion of rare species (Fig. S4). The differences in rare and common relative abundances were most pronounced for large changes in CWC. This was also predicted by a heuristic model that considers the fragmentation of the soil aqueous phase [29] (Fig. S4a). We note that CWC is affected by MAT via potential evapotranspiration that increases with higher temperatures. CWC also covaries with soil pH [17, 29] that is often reported as a key driver of bacterial diversity [28] and species abundance [13] due to the influence of climatic water balance on soil pH [46] (Fig. S3).

### Dry soil reduces the dominance of common bacterial species

**Fig. 4:**
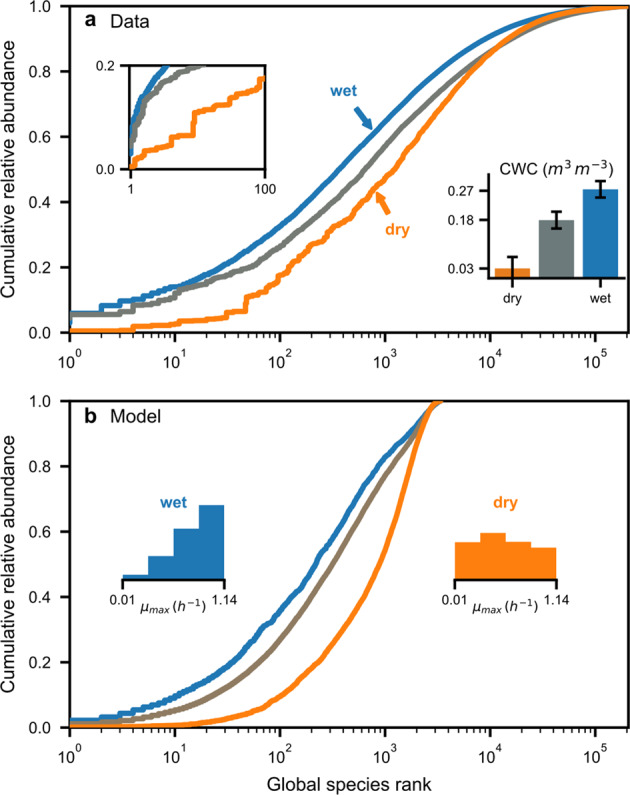
Soil bacterial community shifts with water content—drier conditions suppress common species. **a** The relative abundance distributions (RADs) of soil bacteria for three groups of climatic water contents (CWC; bars indicate mean ± SD) are shown as cumulative relative abundance [17]. Values are sorted by global species rank with one indicating the globally most abundant. The RAD displays a systematic shift toward high ranks under dry conditions. The inset figure on the left shows the 100 most abundant species on a linear scale. **b** The spatially explicit individual-based model (SIM) confirms the observed tendency. The distribution of modeled species maximum growth rates ($\mu _{{\mathrm{max}}}$) at the end of the simulation indicates that physiological differences are equalized under dry conditions, while the increased relative abundance of common species under wet conditions corresponds to higher growth rates.

A distinct shift in the soil bacterial RAD was observed for different (climatic) soil hydration conditions, with a smaller proportion of common species found in dry regions (Fig. 4a). The SIM results suggested that common species were suppressed under dry conditions where their superior physiological traits could not be expressed and their activity was thus equalized with less-fit species [32] (Fig. 4b). Under dry conditions, the simulated bacterial community became more even in terms of the distribution of maximum growth rates and the species RAD [32, 47]. The total number of simulated individuals ranged from 10^3^ to 10^6^ closely following the soil water contents and average kinetic parameters (maximal growth rate and carbon source affinity; Fig. S5). Slow-growing species in the simulated communities were only favored below water contents of 0.1. Assuming that physiological differences between rare and common species caused the observed shifts in community composition, we could expect changes in patterns of soil bacterial rarity across moisture conditions at shorter timescales.

**Fig. 5:**
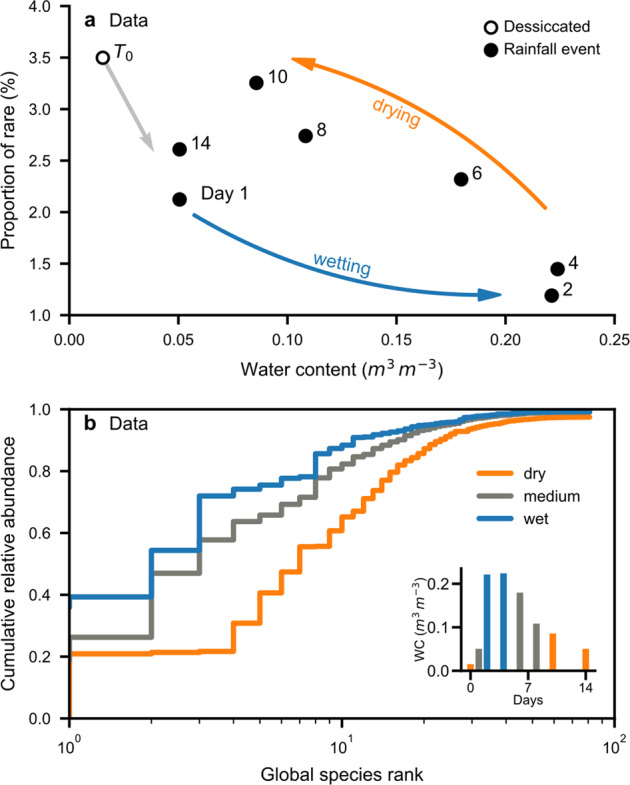
Activity of common and rare soil bacteria shaped by rainfall. **a** Short-term shifts in the proportion of rare species following a winter rain event in the Negev desert (days 1 and 2; *T*_0_ marks full desiccation in summer) with subsequent return to initial community composition as previously reported [37]. Each point represents averaged measurements of bacterial RNA abundance (*n* = 3). **b** Samples were further aggregated to three hydration conditions [37] and are displayed as ranked, cumulative relative abundance. Water contents (WC) at the time of sampling are shown in the inset figure.

To investigate the dynamics of bacterial rarity, we have applied our approach to classifying species RNA using a previously published desert soil community time series [37]. The daily observations were comparable with our simulations and reflect proportions of active bacterial cells from different species[37, 48] . Following a winter rainfall event in the Negev desert, we found that the activity of rare bacterial species dropped during soil wetting and recovered to initial values following soil drying (Fig. 5a). The community displayed consistent shifts where few common species dominated during the unusually wet conditions but were suppressed when the soil was dry (Fig. 5b). We note that the proportion of rare species in this dataset appeared extremely small. This could be partially explained by the taxonomic assignment used, which did not allow to resolve species with very low relative abundances. These “unassigned” species were removed from the analysis and caused the relative abundance to not sum up to unity. More importantly, the RNA-based measurements excluded dormant individuals that could have constituted a large proportion of bacterial communities in dry soils (Fig. 3a).

### Biogeographic patterns of soil bacterial rarity

**Fig. 6:**
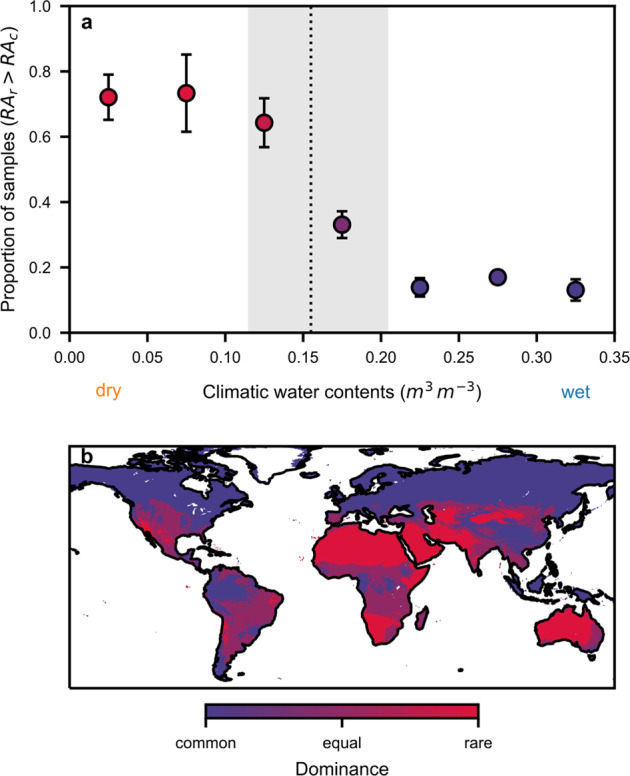
Critical role of soil hydration and the corresponding spatial patterns of rarity. **a** The proportion of samples where the relative abundance (RA) of rare species ($RA_r$) exceeds the relative abundance of common species ($RA_c$) displayed a transition along a gradient of climatic water contents (CWC). Samples were binned by CWC (bin width: 0.05; mean ± SE). The dashed vertical line illustrates the median critical water content below which the aqueous phase is largely disconnected (shading represents the central 95% of global values). **b** Regions based on CWC where rare bacteria are likely to dominate the community RA (red), where common and rare transition (purple), and where the common dominate (blue).

To delineate the geographic bounds where rarity changed, we analyzed community composition concerning CWC in our global dataset. The proportions of soil samples in which rare species jointly dominated community composition (i.e., where the sum of rare relative abundance exceeded 0.5) exhibited a steep transition with CWC (Fig. 6a). We invoked arguments from percolation theory to link the average state of the aqueous phase with its connectivity within soils in different biomes. We have identified a critical water content above which the soil aqueous phase is frequently connected with enhanced opportunities for bacterial interactions and increased carbon fluxes [29]. The critical water content ($\theta _c$) was approximated as 31% [49] of soil porosity [50]. Considering the universal role of water contents in structuring the soil bacterial microbiome and by using only the input data of CWC and soil porosity [51], we mapped global regions where rare bacteria, on average, likely dominated (Fig. 6b). The transition region was represented by the central 95% of global $\theta _c$ values.

## Discussion

In our dataset, only 0.4% of soil bacterial species are consistently classified as common. They are globally more prevalent and more abundant than the majority (99.6%) of species classified as rare. The power of the nonparametric classification of rare and common soil bacteria proposed in this study is the lack of prior assumptions regarding the underlying RAD, which offers general applicability and permits comparison of data from different studies. The classification method is insensitive to “noise” among species with low abundances since it does not contain information that affects threshold selection [33]. Hence, this global classification method consistently labels bacterial species across soils and biomes.

Soil moisture shapes bacterial rarity in agreement with model results of the SIM that demonstrate how the proportions of common species increase toward wetter soils. The large environmental range of common species with high relative abundance is attributed to an intrinsic fitness [23] that enables their global prevalence (Fig. 2d). In wet soils, the bacterial community RADs with high proportions of common species are most similar to the global average RAD and also more closely follow a global ranking of species relative abundance (Fig. S1). This could reflect different bacterial strategies [11] of common species that are enabled under relatively wet conditions. In contrast, the sparse vegetation growing in arid soils limits carbon fluxes and soil-carrying capacity, thereby suppressing the fast growth of a “chosen few” common bacterial species. Dry soils may thus harbor communities with higher evenness, in which species are sheltered in isolated aqueous microhabitats [47] that restrict the ranges of dispersal and likely affect gene flow in these soils [52]. This maintains numerous rare species that, taken together, can contribute to a large proportion of the community. The systematic shifts in RADs with CWC could indicate changing functional diversity of soil bacteria that is linked to specific functional roles [3] and genetic potential [22, 23]. Better attribution of variations in diversity metrics, such as bacterial richness, to environmental factors highlights the apparent sensitivity of rare bacteria that is observed in statistical analysis [17].

A transition in processes governing community assembly [1] has been postulated for decreased environmental “randomness” [53] as expected in wet soils with enhanced aqueous-phase connectedness. On average, a well-connected soil aqueous phase also implies higher fluxes of nutrients and increased bacterial mobility (i.e., a “selection-dominated” regime [53]). In dry soils, the aqueous habitat fragmentation imposes “randomness” (i.e., “drift-dominated” communities [53]). The limitations to cell dispersal further stabilize the spatial distribution of soil bacteria and the patchy access to resources reduces selection by competitive exclusion. Most importantly, reduced nutrient fluxes in dry soils invariably constrain the physiological advantage of fast-growing (common) species in agreement with recent experimental evidence [54]. This is reflected in the results of the SIM by more even distributions of abundances and maximal growth rates under dry conditions (Fig. 4b). Growth-limiting conditions in desert soils cause a large drop of rare species’ proportion when removing inactive cells from the simulation results by excluding cells that did not divide (Fig. 3a). This tendency is consistent with the observed low-activity levels of rare species in desert communities (Fig. 5). We should thus expect many bacterial cells in natural communities to be dormant and at low abundance [21, 48], with particular functional implications for dry soils [10].

The sensitivity of rare species to environmental conditions may be partially explained by a hydration-centered modeling framework without assigning specific functional traits. Nonetheless, rare bacteria constitute a deep reservoir of traits and we can expect their functional contributions to vary with CWC. Broader ecosystem functions, such as soil heterotrophic respiration, are widespread among bacterial species [5] and are likely to be associated with the activity of common species that make up most of the community biomass [24]. This is evidenced, for example, by the rapid saturation of CO_2_ production with increasing bacterial richness in microcosm experiments [4, 14]. The degradation of complex carbon sources, on the other hand, requires the activity of specific enzymes that are thought to be contributed by (metabolically versatile) rare bacteria [3, 5, 7, 19, 55] and could be associated with slower growth compared to mineralization of readily degradable sugars. Additional factors (other than hydration) are likely to contribute to differences in growth between common and rare bacteria. For example, certain environmental factors (e.g., high-salt concentrations and varying redox conditions) that require specific physiological adaptations are currently not explicitly accounted for. This could be particularly relevant for conditionally rare taxa [56], which are implicitly represented in our analysis as members of the common group with relatively low global prevalence (Fig. 2d). Small differences in growth properties could amplify the relative abundance of particular bacterial taxa to extents that make them common across samples. For example, few taxa (γ-Proteobacteria, Clostridia, Bacilli, and Bacteroidia) dominated bacterial activity in a desert community only while the soil was wet [37]. This short period was enough to propel these taxa to prominence [10], causing them to be labeled as “common” across samples. Since rainfall events in deserts are very infrequent and offer short time windows of opportunity, we do not expect many globally common species to be detected in this biome and observe that rare species are on average seven times more numerous in regions characterized by low rainfall frequencies relative to common species (Fig. 2e). Processes at scales smaller than considered by the rainfall data (e.g., anoxic microsites [37]) could contribute to the high variability of observed relations. Nonetheless, the general tendencies are in agreement with observations on rare soil bacterial diversity where time-sensitive conditionally rare taxa contributed to changes in community structure, but could not account for the observed whole-community variability, the latter being under the control of spatiotemporal abundance fluctuations of common species that do not cross the threshold to rarity [57].

Our procedure delineates common species and successfully captured dynamics of soil bacterial community activity that could be manifested in RADs under longer timescales. Previous statistical analysis supports a pivotal role of CWC in shaping natural soil bacterial communities [17] and points to the variable’s importance for disentangling the effects of other environmental factors (e.g., carbon input, temperature, and soil pH) that are reconciled in the context of biome-specific hydration conditions and carrying capacity (Fig. S5). Carrying capacity increases for lower temperatures by reducing maintenance and growth rates [58], but shifts caused by temperature are much smaller compared to those caused by soil hydration conditions, in agreement with observations on the global drivers of soil microbial carbon [31]. We distinguish environments in which bacterial species abundance is shaped primarily by physical constraints with limited biomass production (fragmented aqueous habitats under dry conditions) from environments where physiological traits could shape community composition (enhanced nutrient fluxes under wet conditions) [25, 53]. We thus suggest future sampling efforts to concentrate on drier and underrepresented ecosystems that are crucial for quantifying the functional consequences of climatic shifts for bacterial rarity. Regions dominated by the rare soil microbiome with high sensitivity to environmental factors (Fig. 6b) could harbor large functional potential that is readily expressed under variations in climatic conditions [10].

## Supplementary information

Supplementary Information

## Data Availability

The datasets generated and/or analyzed during the current study are available from the corresponding author on reasonable request.
