# Supplementary material for: The chosen few—variations in common and rare soil bacteria across biomes
Source: ISME J. 2021 May 25;15(11):3315–25. doi: 10.1038/s41396-021-00981-3 (PMC8528968; doi:10.1038/s41396-021-00981-3)
Supplement: Supplementary file 1 — Supplementary Information [file 41396_2021_981_MOESM1_ESM.pdf]

Supplementary information for:

The chosen few – variations in common and rare soil bacteria across biomes

Authors

Samuel Bickel[1,2*] and Dani Or[1,3]

Affiliation

[1] Soil, Terrestrial and Environmental Physics (STEP); Institute of Biogeochemistry and Pollutant dynamics (IBP); Swiss Federal Institute of Technology (ETH), Zürich

[2] Physics of Soils and Terrestrial Ecosystems (POSE), Institute of Terrestrial Ecosystems (ITES), Swiss Federal Institute of Technology (ETH), Zürich

[3] Division of Hydrologic Sciences; Desert Research Institute, Reno, NV, USA

* Corresponding author: SB (https://orcid.org/0000-0002-9839-4591)

Content

Supplementary Methods	p. 2-3
Supplementary Figures	p. 4-8
Supplementary Tables	p. 9-10
References	p. 11

Supplementary Methods

Multispecies growth in the spatially-explicit individual-based model (SIM)

We simulate soil moisture-dependent growth and motility of multispecies soil bacterial communities that rely on shared and diffusible resources [1–3]. The modeled species are represented by unique combinations of kinetic (Monod type) parameters that reflect the competitive ability under locally variable carbon source concentrations. Predictions of the SIM are evaluated for a range of soil moisture conditions that shape diffusion from heterogeneous carbon sources via water films on hydrated soil surfaces and by considering the temperature dependency of the kinetic parameters.

We modify our previous model implementation [1] (https://doi.org/10.5281/zenodo.3558542) allowing multiple nutrients ("carbon sources") to be consumed by different species. Each species i is represented by a set of Monod parameters (maximal growth rate μ_{max} and the half-saturation constant K) for three carbon modeled sources j. This results in three sets of parameters per species $(\mu_{max,j}\ K_j)_i$. Considering four steps for the discretization of the parameter space this resulted in R = 3 360 species (possible permutations of parameter pairs).

The partitioning of growth capacity is loosely based on a previously developed model [4] generalized for an arbitrary number of nutrients. The sub-additive growth rate of a species depending on carbon source concentration C_j is given by equation 1:

$$\mu_i = \frac{\sum_j \mu_{max,i,j}\chi_{i,j}}{(1+\sum_j \alpha_{i,j}\chi_{i,j})} \text{ with } \chi_{i,j} = \frac{C_j}{K_{i,j}+(1-\alpha_{i,j})C_j} \text{ and } \alpha_{i,j} = \frac{\mu_{max,i,j}}{\sum_j \mu_{max,i,j}} \quad (1)$$

For each species i, and carbon source j the coefficient $\chi_{i,j}$ describes the concentration dependency of growth rate μ_i that is related to the distribution of $\mu_{max,i,j}$ by the weights $\alpha_{i,j}$. This formulation is well-bounded and ensures that specialist species (with $\alpha_{i,j}$ close to one) can grow at the rates of single substrates. The partitioning of cellular capacity (e.g., cell surface area for nutrient absorption) is described by normalizing Monod coefficients using equation 2:

$$\kappa_{i,j} = \frac{M_{i,j}}{\sum_j M_{i,j}} \text{ with } M_{i,j} = \frac{C_j}{K_{i,j}+C_j} \quad (2)$$

This leads to the definition of species-specific cellular maintenance rate $k_{m,i}$ that is a weighted fraction (f_m = 0.01) of maximal, nutrient-specific growth rates described by equation 3:

$$k_{m,i} = f_m \sum_j \kappa_{i,j}\mu_{max,i,j} \quad (3)$$

The change in cell mass Δm_i for an individual of species i over time Δt is given by equation 4:

$$\Delta m_i = (\mu_i - k_{m,i})m_i\Delta t \quad (4)$$

Resource utilization $r_{i,j}$ is assigned a constant yield Y across species and nutrients (equation 5). The changes in the mass of nutrients Δm_j vary spatially and depend on the volume of water V_w at a particular location. Bacteria respond to environmental concentrations C_j but their growth is limited by the mass of carbon m_j that is locally available for consumption. Hence, the diffused carbon

concentrations C_j are converted using V_w ($m_j = C_j V_w$) and cells cannot take up more carbon than would be available locally (within Δt).

$$r_{i,j} = -\frac{\kappa_{i,j}\mu_i m_i}{Y} \quad (5)$$

$$\Delta m_j = \sum_{i=1}^{R} r_{i,j}\,\Delta t \quad (6)$$

For simulations with temperature dependency, two additional factors based on the Schoolfield [5] model are used that reduce maximum growth and maintenance rates ($f_\mu(T)$ and. $f_k(T)$, respectively). The factor $f_k(T)$ affecting maintenance rates was assumed constant above the optimum temperature (unlike $f_\mu(T)$ that drops sharply above optimum temperature) but was otherwise identical. Parameters used for the temperature dependency were obtained from a recent study [6]. The Schoolfield [5] model was fitted to published data [6] of different species to obtain a single, average temperature response curve that was normalized to the maximal rate (the rate at an optimal temperature T_{opt}). Parameters used were: energy of activation (E_a = 0.4 ± 0.1 eV), energy of inactivation (E_h = 2.0 ± 0.1 eV) and temperature of inactivation (T_h = 31 ± 2 °C) that result in T_{opt} = 27 °C, which marks the temperature of the SIM without explicit temperature dependency. In simulations of biomes with subzero MAT, a temperature of 0 °C was used instead of the biome average MAT. The trait parameter values are summarized in Table S1.

Supplementary Figures

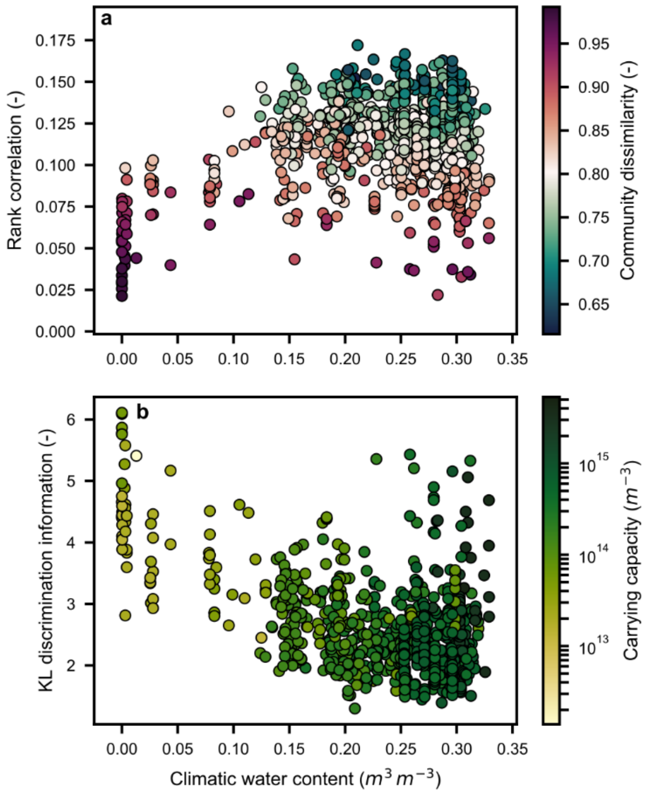

Figure S1 | Shifts in community composition with climatic water contents relative to the global average relative abundance distribution (RAD). To detect changes in community composition with CWC, all sample RADs were compared to the global RAD. Spearman rank correlation and Bray-Curtis dissimilarity were used to measure changes in species relative abundance ranking and community composition, respectively. **a**, Spearman rank correlation between each sample's RAD and the global RAD. Colors indicate Bray-Curtis community dissimilarity. **b**, Discrimination information measured via Kullback-Leibler (KL) divergence between each sample RADs and the global RAD. The amount of information gained from the RAD of individual samples relative to the global RAD displayed a consistent decrease with increasing CWC as was previously postulated for reduced environmental heterogeneity [7]. Lower values indicate that less information is needed to describe the samples' RAD relative to the global RAD; i.e., similar species are present at similar abundances. Colors indicate estimated cell density at carrying capacity [1].

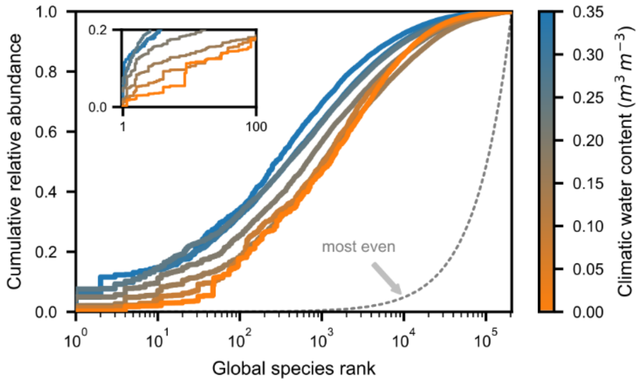

Figure S2 | Gradual shift in community composition with varying climatic water contents. The relative abundance distributions (RADs) of soil bacteria for groups of climatic water contents (bins of 0.05 resolve the gradual change shown in the data of Fig. 4a) and are shown as cumulative relative abundance using previously published data [8]. Values are sorted by global species rank that ranges from most (rank one) to least abundant. The distribution displays a systematic shift towards an increased proportion of rare species under dry conditions. The distribution is shifted towards more even soil bacterial communities where each species would contribute equally to community composition regardless of their global ranks (dashed line). The inset figure on the left shows the 100 most abundant species on a linear scale.

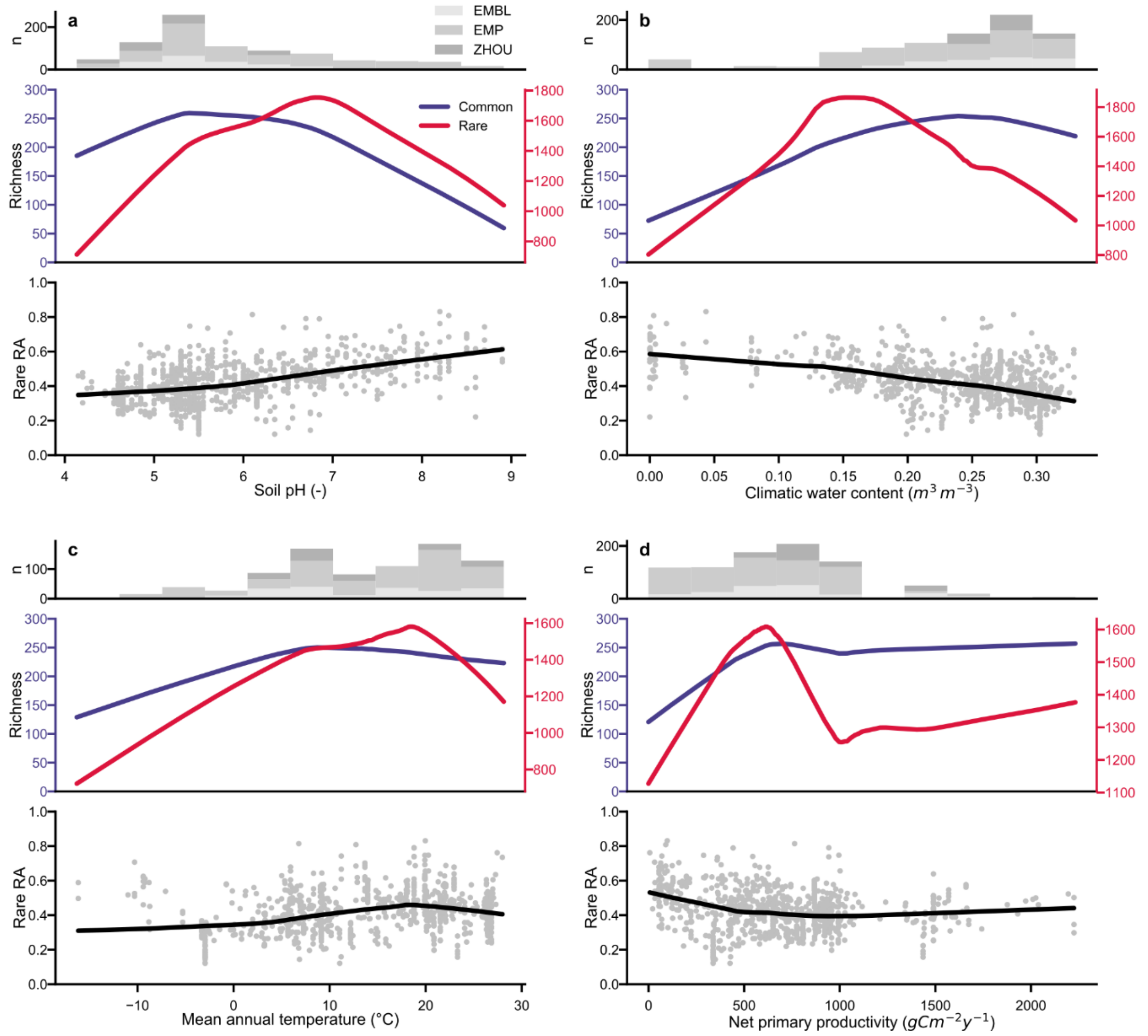

Figure S3 | The proportion of rare bacteria varies with environmental conditions. a-d Empirical trends in relative abundance (RA) of rare bacteria compared for selected variables (grey symbols and solid black line, individual samples (n=844, from studies EMBL [9], EMP [10] and ZHOU [11]) and locally weighted scatter plot smooth (LOWESS). Colored lines indicate the LOWESS of bacterial richness for common and rare bacteria (purple and red, respectively). Rare and common species are indicated on the separate y-axis. **a**, soil pH, **b**, climatic water contents, **c**, mean annual temperature, and **d**, net primary productivity.

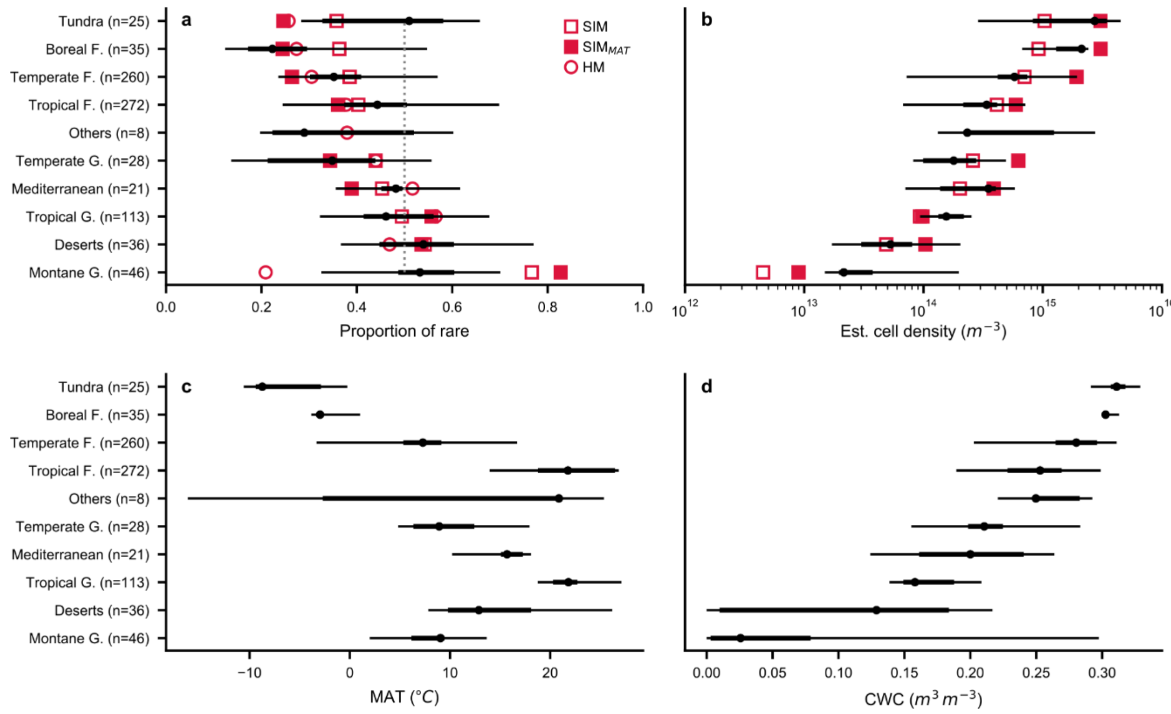

Figure S4 | Simulated bacterial community for different biomes. a-d, Median and central 50% and 95% of values are indicated for each biome. Predictions by a heuristic model [1] (HM, open circles) are shown together with model results of the SIM, and SIM with temperature dependency (SIM$_{MAT}$; open and closed squares, respectively). **a**, Proportion of rare species. **b**, Carrying capacity (estimated cell density). **c**, Mean annual temperature (MAT) and **d**, climatic water content (CWC) that provide average input values for the SIM.

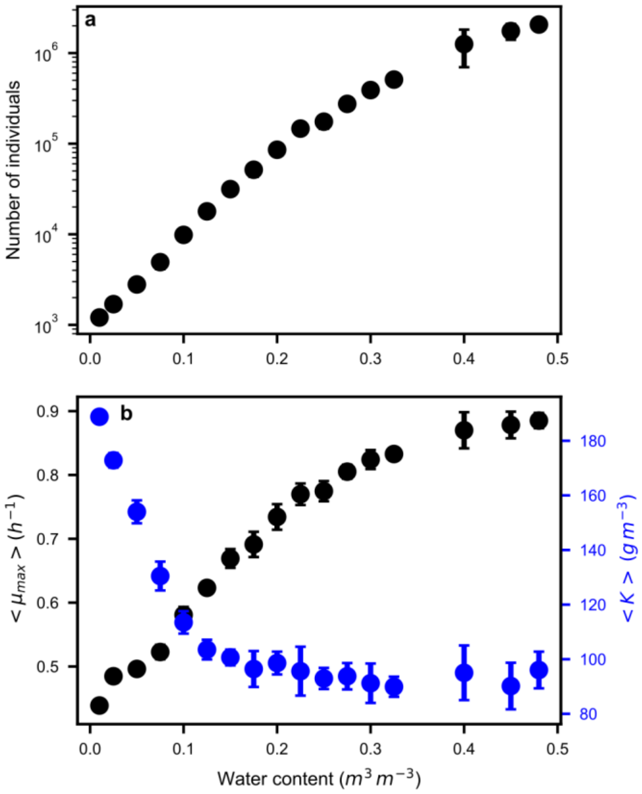

Figure S5 | Simulated bacterial community for varying water contents. a, The number of individuals increases exponentially with water contents although some saturation can be observed for very wet conditions (mean ± SD, n = 5 except for water contents > 0.35 where n = 3). The number of potentially maintained individuals is not prescribed and emerges from increased fluxes at high water contents, carbon input (boundary conditions), and physiological properties of individual species. **b**, Ensemble averages of Monod parameters (μ_{max} and K, maximal growth rate and half-saturation constant, respectively) display consistent patterns with water contents.

Supplementary Tables

Table S1 | Trait parameters used in the spatially-explicit individual-based model (SIM). The physiological parameters that describe traits of different species and their dependency on temperature are listed with their respective values and units.

Parameter	Symbol	Unit	Value(s)
Maximal growth rate	μ_{max}	$\mathrm{h^{-1}}$	0.01, 0.39, 0.76, 1.14
Half saturation constant	K	$\mathrm{g\ m^{-3}}$	1, 9, 77, 680
Maintenance fraction	f_m	$-$	0.01
Biomass yield	Y	$\mathrm{g\ g^{-1}}$	0.5
Energy of activation	E_a	eV	0.4 ± 0.1
Temperature of inactivation	T_h	°C	31 ± 2
Optimal temperature	T_{opt}	°C	27

Table S2 | Resampling of the dataset to test the robustness of threshold selection. A subset of samples was selected randomly to perform resampling with replacement (n_{boot} = 1’000) from the previously published data [8] on community composition. The threshold *t* of relative abundance that is used to distinguish rare and common species and the resulting relative abundance of rare bacteria RA_r are shown for the resampled datasets. Mean and SD are reported.

**Samples**	t_{mean} **(%)**	t_{SD} **(%)**	$RA_{r,mean}$ **(%)**	$RA_{r,SD}$ **(%)**
52	0.021	0.003	39	11
105	0.021	0.002	41	12
211	0.021	0.002	42	12
422	0.020	0.002	42	12
844	0.019	0.002	42	12

References

1. Bickel S, Or D. Soil bacterial diversity mediated by microscale aqueous-phase processes across biomes. *Nat Commun* 2020; **11**: 1–9.
2. Wang G, Or D. Hydration dynamics promote bacterial coexistence on rough surfaces. *ISME J* 2013; **7**: 395–404.
3. Kim M, Or D. Individual-based model of microbial life on hydrated rough soil surfaces. *PLOS ONE* 2016; **11**: 1–31.
4. Hermsen R, Okano H, You C, Werner N, Hwa T. A growth-rate composition formula for the growth of E.coli on co-utilized carbon substrates. *Mol Syst Biol* 2015; **11**: 1–6.
5. Schoolfield RM, Sharpe PJH, Magnuson CE. Non-linear regression of biological temperature-dependent rate models based on absolute reaction-rate theory. *J Theor Biol* 1981; **88**: 719–731.
6. García FC, Bestion E, Warfield R, Yvon-Durocher G. Changes in temperature alter the relationship between biodiversity and ecosystem functioning. *Proc Natl Acad Sci* 2018; **115**: 10989–10994.
7. Fisher CK, Mehta P. The transition between the niche and neutral regimes in ecology. *Proc Natl Acad Sci* 2014; **111**: 13111–13116.
8. Bickel S, Chen X, Papritz A, Or D. A hierarchy of environmental covariates control the global biogeography of soil bacterial richness. *Sci Rep* 2019; **9**: 1–10.
9. Bahram M, Hildebrand F, Forslund SK, Anderson JL, Soudzilovskaia NA, Bodegom PM, et al. Structure and function of the global topsoil microbiome. *Nature* 2018; **560**: 233–237.
10. Thompson LR, Jex AR, Campbell AH, Linz AM, Berry A, Williams AE, et al. A communal catalogue reveals Earth's multiscale microbial diversity. *Nature* 2017; **551**: 457–463.
11. Zhou J, Deng Y, Shen L, Wen C, Yan Q, Ning D, et al. Temperature mediates continental-scale diversity of microbes in forest soils. *Nat Commun* 2016; **7**: 1–10.
